# Supplementary material for: Baicalein Inhibits Benzo[a]pyrene-Induced Toxic Response by Downregulating Src Phosphorylation and by Upregulating NRF2-HMOX1 System
Source: Antioxidants (Basel). 2020 Jun 9;9(6):507. doi: 10.3390/antiox9060507 (PMC7346154; doi:10.3390/antiox9060507)
Supplement: Supplementary file 1 [file antioxidants-09-00507-s001.pdf]

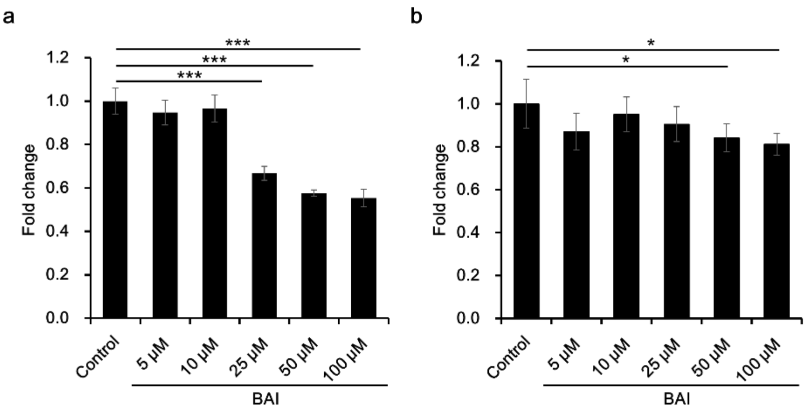

Supplementary Figure S1: Cell viability of BAI-treated keratinocytes.

(**a**) NHEKs and (**b**) HaCaT cells were treated with DMSO (0.1%) or BAI (5, 10, 25, 50 or 100 μM) for 24 h and cell viability was measured using a formazan-based viable cell counting method. Experiments were repeated three times. Mean ± SD are shown. **P* < 0.05 and ****P* < 0.001.

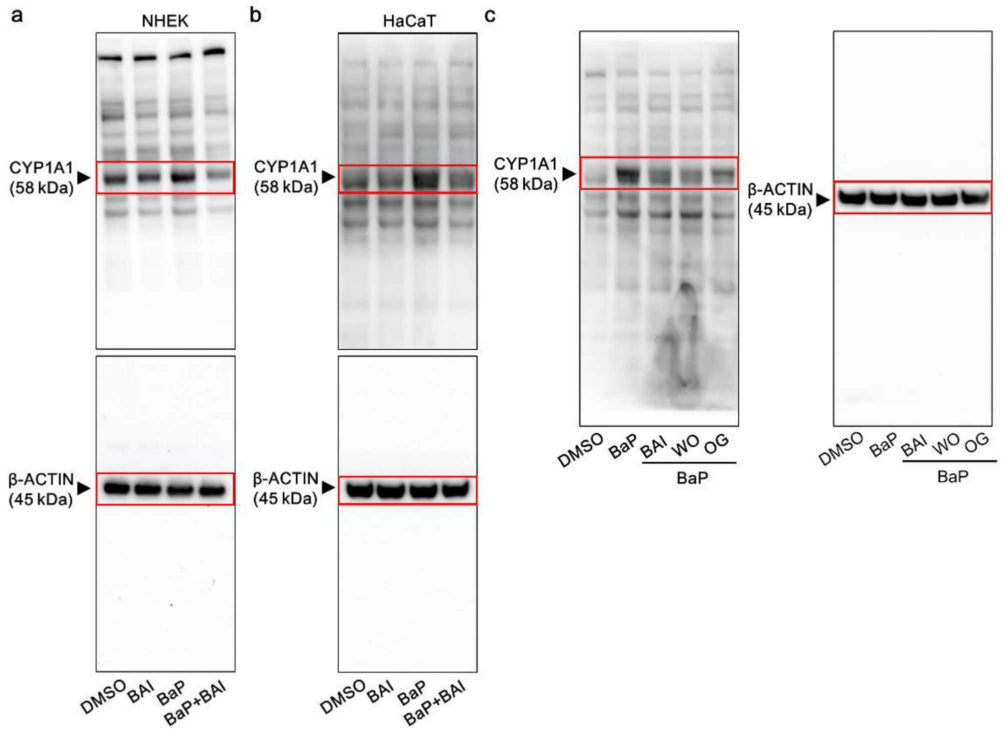

Supplementary Figure S2: Uncropped images of western blotting corresponding to Figure 2.

(**a**) Uncropped images of Figure 2a, (**b**) Figure 2b, and (**c**) Figure 2c. β-actin at the same run was used as loading control. The red boxes indicate the cropped area shown in main Figure.

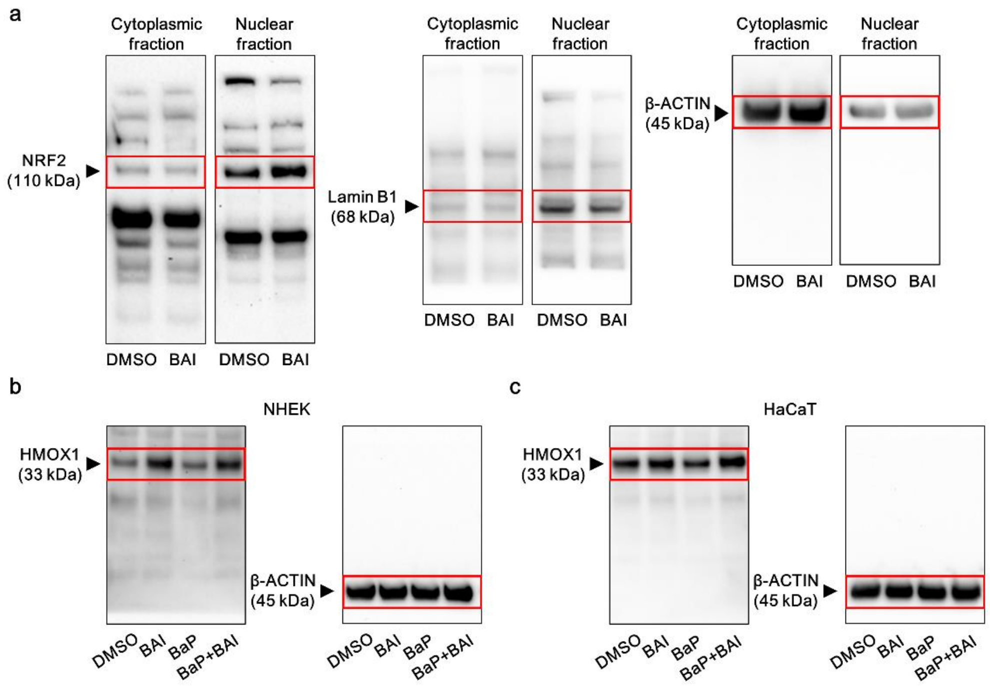

Supplementary Figure S3: Uncropped images of western blotting corresponding to Figure 3 and 4.

(**a**) Uncropped images of Figure 3. Lamin B1 and β-actin at the same run was used as loading control of nuclear and cytoplasmic fraction, responsively. (**b** and **c**) Uncropped images of (**b**) Figure 4a, and (**c**) Figure 4b. β-actin at the same run was used as loading control. Membranes were cut at approximately 40 kDa to separately probe HMOX1 and β-actin on the same membrane/run. The red boxes indicate the cropped area shown in main Figure.

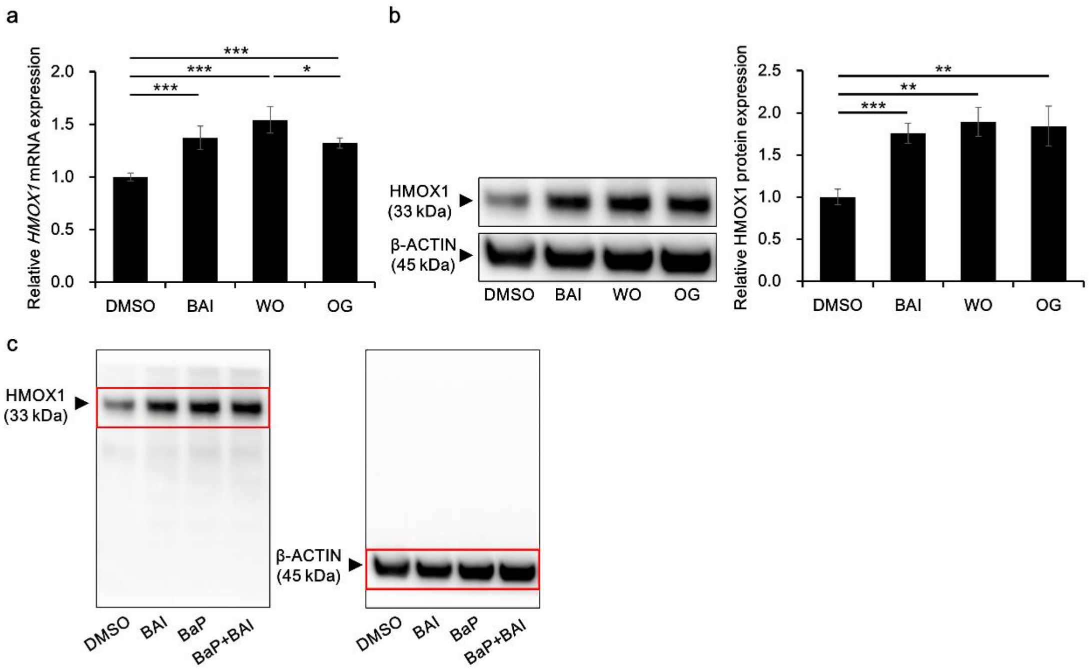

Supplementary Figure S4: BAI-containing crude/herbal drug induces HMOX1 expression in keratinocytes.

(**a**) HaCaT cells were treated with DMSO (0.1%), BAI (25 μM), WO (100 μg/mL) or OG (100 μg/mL) for 6 h and assessed for *HMOX1* mRNA expression. (**b**) HaCaT cells were treated as (**a**) for 24 h and assessed for HMOX protein expression. Experiments were performed in triplicate wells and repeated three times. Mean ± SD are shown. **P* < 0.05, ***P* < 0.01, and ****P* < 0.001. (**c**)Uncropped images of (**b**). β-actin at the same run was used as loading control. Membranes were cut at approximately 40 kDa to separately probe HMOX1 and β-actin on the same membrane/run. The red boxes indicate the cropped area shown in Supplementary Figure S4b.

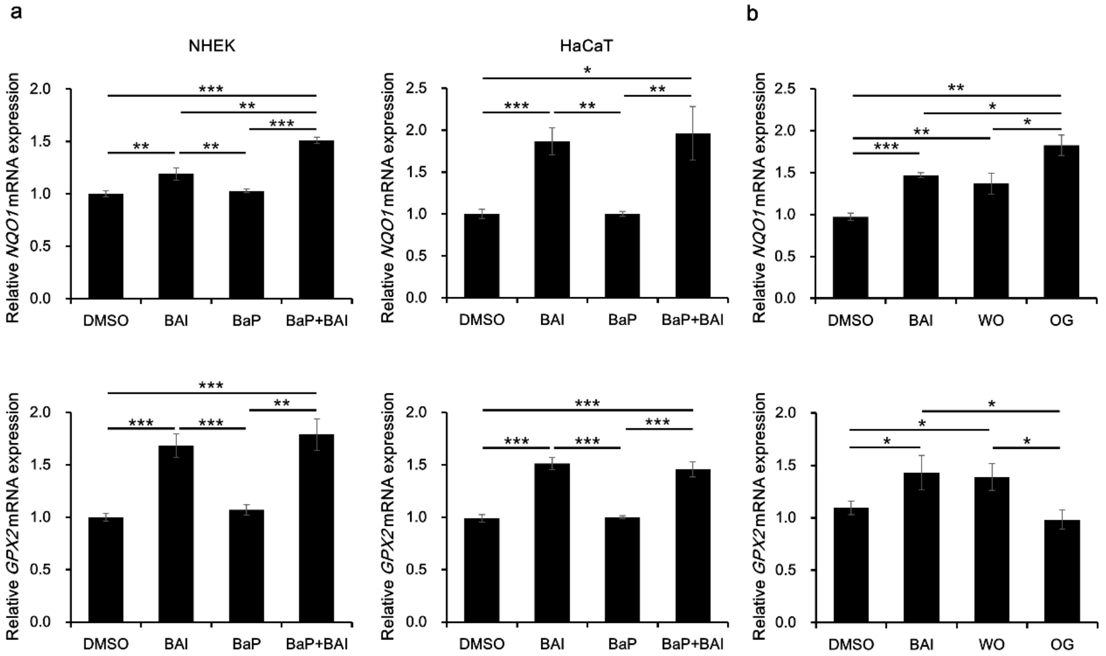

Supplementary Figure S5: BAI and BAI-containing crude/herbal drugs induce the expression of antioxidative genes downstream of NRF2 pathway.

(**a**) NHEKs and (**b**) HaCaT cells were treated with DMSO (0.1%), BAI (10 μM for NHEKs and 25 μM for HaCaT cells), BaP (1 μM) or combination of BaP and BAI for 6 h and assessed for the expressions of *NQO1* and *GPX2*. (**c**) HaCaT cells were treated with DMSO (0.1%), BAI (25 μM), WO (100 μg/mL) or OG (100 μg/mL) for 6 h and assessed for the expressions of *NQO1* and *GPX2*. Experiments were performed in triplicate wells and repeated three times. Mean ± SD are shown. **P* < 0.05, ***P* < 0.01, and ****P* < 0.001.

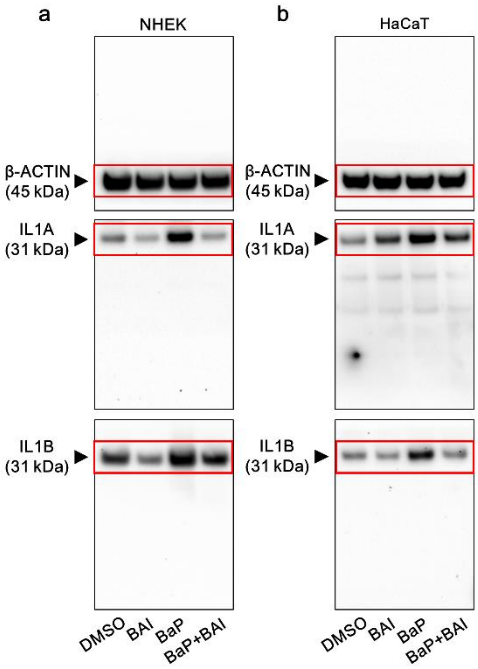

Supplementary Figure S6: Uncropped images of western blotting corresponding to Figure 6.

Uncropped images of (**a**) Figure 6c and (**b**) Figure 6d. β-actin at the same run was used as loading control. Membranes were cut at approximately 40 kDa to separately probe IL1A, IL1B, and β-actin on the same membrane/run. The red boxes indicate the cropped area shown in main Figure.

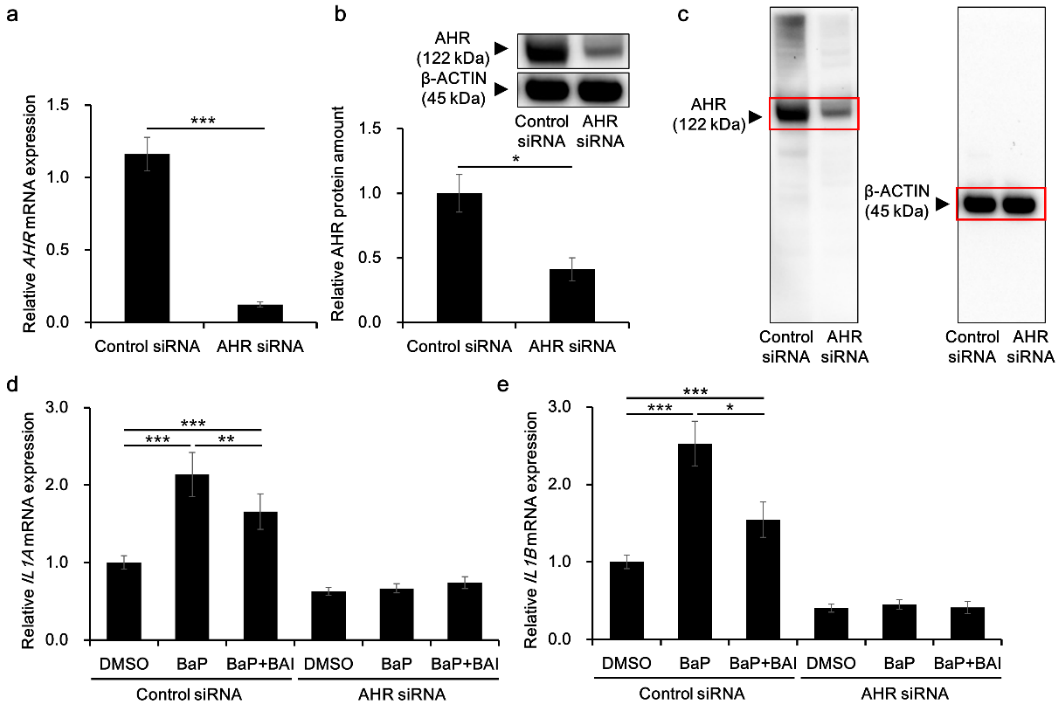

Supplementary Figure S7: BaP-mediated IL1A and IL1B induction is dependent on AHR.

HaCaT cells were transfected with control or AHR siRNA for 48 h and knockdown efficiency was assessed at (**a**) the mRNA level by qRT-PCR and (**b**) the protein level by western blotting. (**c**) Uncropped images of (**b**). β-actin at the same run was used as loading control. (**d** and **e**) siRNA-transfected cells were further treated with DMSO (0.1%), BaP (1 μM) or a combination of BaP and BAI (25 μM) and the expression of (**d**) *IL1A* and (**e**) *IL1B* was assessed at 6 h after treatment. Each experiment was performed in triplicate wells and repeated three times. Mean ± SD are shown. *$P < 0.05$, **$P < 0.01$ and ***$P < 0.001$. The red boxes indicate the cropped area shown in Supplementary Figure S7b.

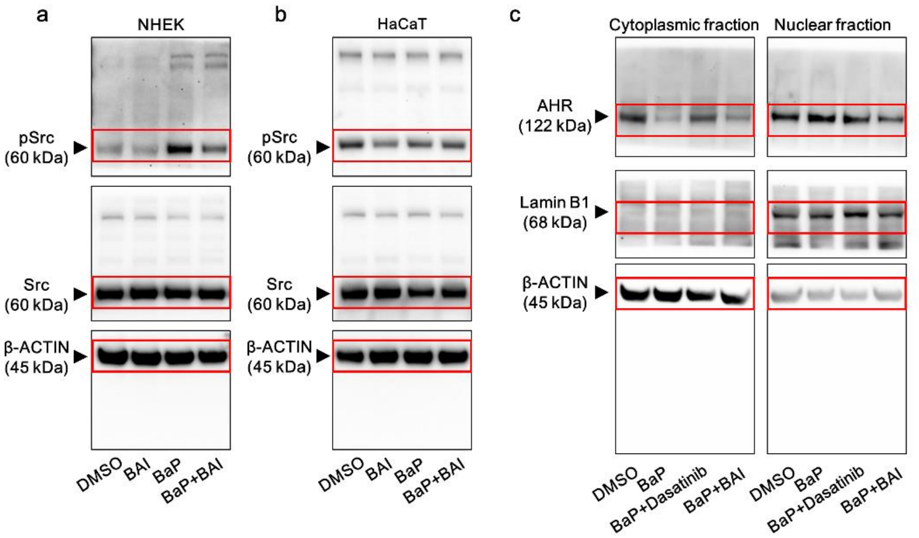

Supplemental Figure S8: Uncropped images of western blotting corresponding to Figure 7 and 8.

(**a**) Uncropped image of Figure 7a and (**b**) Figure 7b. Membranes were cut at 50 kDa to separately probe Src and β-actin on the same membrane/run. (**c**) Uncropped image of Figure 8b. Lamin B1 and β-actin at the same run was used as loading control of nuclear and cytoplasmic fraction, responsively. Membranes were cut at approximately 100 and 50 kDa to separately probe AHR, Lamin B1, and β-actin on the same membrane/run. The red boxes indicate the cropped area shown in main Figure.
